# Exploring the multi-level regulation of lignocellulases in the filamentous fungus *Trichoderma guizhouense* NJAU4742 from an omics perspective

**DOI:** 10.1186/s12934-022-01869-3

**Published:** 2022-07-16

**Authors:** Yanwei Xia, Jingfan Wang, Chuanxu Guo, Huanhuan Xu, Wei Wang, Mingzhu Yang, Qirong Shen, Ruifu Zhang, Youzhi Miao

**Affiliations:** 1 Jiangsu Provincial Key Lab for Organic Solid Waste Utilization, National Engineering Research Center for Organic-based Fertilizers, Jiangsu Collaborative Innovation Center for Solid Organic Waste Resource Utilization, Nanjing Agricultural University, 210095 Nanjing, People’s Republic of China; 2 Key Laboratory of Microbial Resources Collection and Preservation, Ministry of Agriculture, Institute of Agricultural Resources and Regional Planning, Chinese Academy of Agricultural Sciences, 100081 Beijing, China

**Keywords:** Plant biomass degradation, Lignocellulases, Regulatory mechanisms, *Trichoderma*

## Abstract

**Background:**

Filamentous fungi are highly efficient at deconstructing plant biomass by secreting a variety of enzymes, but the complex enzymatic regulation underlying this process is not conserved and remains unclear.

**Results:**

In this study, cellulases and xylanases could specifically respond to Avicel- and xylan-induction, respectively, in lignocellulose-degrading strain *Trichoderma guizhouense* NJAU4742, however, the differentially regulated cellulases and xylanases were both under the absolute control of the same TgXyr1-mediated pathway. Further analysis showed that Avicel could specifically induce cellulase expression, which supported the existence of an unknown specific regulator of cellulases in strain NJAU4742. The xylanase secretion is very complex, GH10 endoxylanases could only be induced by Avicel, while, other major xylanases were significantly induced by both Avicel and xylan. For GH10 xylanases, an unknown specific regulator was also deduced to exist. Meanwhile, the post-transcriptional inhibition was subsequently suggested to stop the Avicel-induced xylanases secretion, which explained the specifically high xylanase activities when induced by xylan in strain NJAU4742. Additionally, an economical strategy used by strain NJAU4742 was proposed to sense the environmental lignocellulose under the carbon starvation condition, that only slightly activating 4 lignocellulose-degrading genes before largely secreting all 33 TgXyr1-controlled lignocellulases if confirming the existence of lignocellulose components.

**Conclusions:**

This study, aiming to explore the unknown mechanisms of plant biomass-degrading enzymes regulation through the combined omics analysis, will open directions for in-depth understanding the complex carbon utilization in filamentous fungi.

**Supplementary Information:**

The online version contains supplementary material available at 10.1186/s12934-022-01869-3.

## Background

Plant biomass, as the most abundant natural material on Earth, can be utilized as a carbon source by microbes, in which filamentous fungi are considered as the outstanding degraders for their ability to secrete all of the necessary enzymes. Turnover of plant biomass by filamentous fungi is an important ecosystem process [1], as well as a key biotechnology that has been harnessed industrially to convert plant biomass into biofuels or other high-value compounds [2]. In nature, plant biomass is composed mainly of lignocellulose, which contains cellulose (40-50%), hemicellulose (25-30%) and lignin (15-30%) [3]. Cellulose is the most abundant polysaccharide, and exists mainly as the recalcitrant crystalline structure formed by the β-1,4-linked D-glucose polymeric chains. Hemicellulose includes polysaccharides with β-1,4-linked backbones, such as xylan, xyloglucan and mannan. Lignin, adding rigidity to the plant biomass, is composed of polymers of aromatic residues and thus very recalcitrant to enzymatic degradation. There are approximately 100–1000 carbohydrate-active enzyme genes (CAZyme genes) in different filamentous fungal genomes [4]. Plant biomass-degrading enzymes (PBDEs) mainly come from the groups of glycoside hydrolases (GHs), carbohydrate esterases (CEs), polysaccharide lyases (PLs) and auxiliary activities (AAs) in the CAZyme genes [5]. These enzymes include cellulases, xylanases, mannanases, ligninases, etc., which can be further subdivided into many detailed enzymes acting on the backbone or side chains of polysaccharides, thereby synergistically degrading the whole complex structure of lignocellulose [6].

The mechanism by which filamentous fungi regulate PBDEs secretion has always been a research hotspot, and many regulators have been identified from various fungi. CreA/CRE1, mediating carbon catabolite repression (CCR), is conserved in filamentous fungi and represses nearly all PBDEs when simple carbon sources (glucose, sucrose, etc.) are available [7]. In *Neurospora crassa*, cellulases are mainly controlled by two positive regulators of CLR-1 and CLR-2. CLR-1 is responsible for activating CLR-2, which further activates the downstream cellulase genes [8]. Under non-inducing conditions, the CLR-3 regulator acts through CLR-1 to repress the cellulolytic response [9]. Meanwhile, the hyperosmotic response (OS) pathway can also repress PBDE genes when sensing extracellular high osmolarity of soluble sugars, which is not overlapped with the CreA/CRE1 mediated CCR [9]. In addition, regulators of VIB1, CLR-4 and COL26 have also been reported to directly or indirectly affect cellulolytic genes expression in *N. crassa* [10, 11]. In another model filamentous fungus, *Trichoderma reesei*, there is a significantly different system for PBDEs regulation [12], in which cellulase and hemicellulase genes are mainly positively regulated by Xyr1. CLR-1 and CLR-2 homologs exist but do not function in cellulases regulation of *T. reesei* [13]. There are also 7 other transcription factors identified as responsible for the regulation of different enzymes in *T. reesei*. For example, XPP1 (Xylanase promoter-binding protein 1) inhibits xylanase expressions in the culture broth during the later stages of fermentation [14]. RCE1 inhibits cellulase genes by competitively binding with Xyr1 to their promoters. ACE3 can specifically bind to the promoters of cellulase and Xyr1 genes, thereby activating extracellular cellulase activities [15]. Among other regulators in *T. reesei*, ACE1 is a negative regulator [16], while HAP2/3/5, ACE2 and RXE1 are positive regulators [17–19]. The above systems of PBDEs regulation in *N. crassa* and *T. reesei* have their own conserveness in filamentous fungi, showing the population differentiation. However, some questions remain, e.g., how diverse are the PBDEs regulatory mechanisms? How are the complex networks of regulating different PBDEs in filamentous fungi?

The *Trichoderma guizhouense* NJAU4742, an efficient lignocellulose degrader and plant beneficial fungus [20–22], was mass-produced by the solid fermentation mainly using crop straws as the carbon source in biofertilizer area of China. Thus, in this study, strain NJAU4742 was used to investigate the regulation of PBDEs when induced by different polysaccharides or under carbon starvation. Through the genetic manipulation, enzymology and omics analysis, we initially revealed the multi-level regulation of lignocellulases, especially for cellulases and xylanases, which were mediated by unknown specific regulators and the post-transcriptional inhibitory effects. The inferred network of lignocellulases regulation in this study will guide the subsequent exploration of molecular mechanisms and provide more target sites for genetic modification in filamentous fungi.

## Results

### Significant differences between Avicel- and xylan-induced extracellular proteins in *Trichoderma guizhouense* NJAU4742

**Fig. 1:**
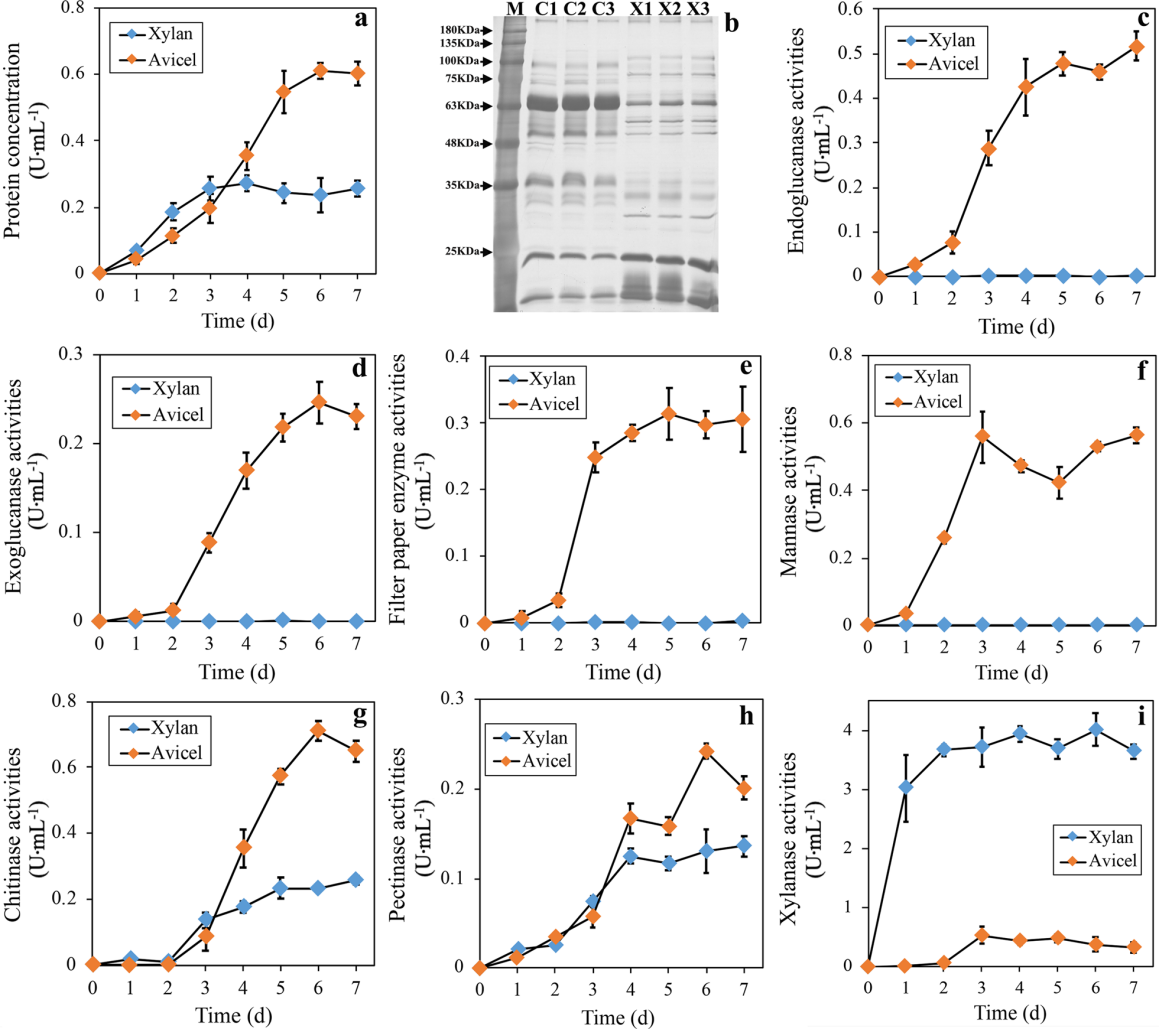
SDS-PAGE analysis and enzyme activity determination for Avicel/xylan-induced extracellular proteins (AIEP/XIEP). The mycelium of strain NJAU4742 was incubated in MM medium containing Avicel or xylan. During the 7 days, extracellular protein concentration (**a**), SDS-PAGE of AIEP/XIEP at the fifth day (**b**), endoglucanase activities (**c**), exoglucanase activities (**d**), filter paper enzyme activities (**e**), mannanase activities (**f**), chitinase activities (**g**), pectinase activities (**h**) and xylanase activities (**i**) were detected. AIEP/XIEP on the fifth day were extracted and then separated using the SDS-PAGE followed by silver staining. M stands for the protein marker; C1-3 an X1-3 stand for the three biological replicates of AIEP and XIEP, respectively. Each detection had three replicates

Filamentous fungi can release soluble sugars from the plant biomass by secreting various enzymes, which include cellulases, hemicellulases and ligninases. To investigate the secretion characteristics of these enzymes in *T. guizhouense* NJAU4742, we performed the cultivation using cellulose (Avicel) and xylan, two main polysaccharides in plant biomass, as the sole carbon source respectively. In this process, extracellular protein concentrations, different enzyme activities and SDS-PAGE results were detected and shown in Fig. 1. Avicel-induced extracellular proteins (**AIEP**, stable at 0.59 ± 0.03 mg‧mL^− 1^) had significantly higher concentration (2.32-fold, *p* < 0.01) than xylan-induced extracellular proteins (**XIEP**, stable at 0.25 ± 0.01 mg‧mL^− 1^) after the fifth incubation day (Fig. 1a). SDS-PAGE profiles showed the significant differences in bands number and staining depth between AIEP and XIEP, and protein bands at approximately 63 KDa contributed strongly to total amount of AIEP (Fig. 1b). Enzyme activity detection showed that cellulase activities, including endoglucanases, exoglucanases and FPAases, and mannanase activities existed only in AIEP (Fig. 1c and f). Chitinase and pectinase activities were significantly higher in AIEP than in XIEP (Fig. 1g, h, p < 0.01). For xylanase activities, XIEP had a stable value of approximately 3.84 ± 0.16 (U‧mL^− 1^), while AIEP had a low value of only 0.43 ± 0.08 U‧mL^− 1^ (Fig. 1i, p < 0.01). These results demonstrate the significant differences in detail between AIEP and XIEP in *T. guizhouense* NJAU4742. As the main polysaccharide-degrading enzymes, cellulases were specifically detected in AIEP, while, xylanases were mainly secreted in XIEP.

### Proteomic identification and comparison of Avicel- and xylan-induced extracellular proteins in *Trichoderma guizhouense* NJAU4742

To further distinguish the differences between AIEP and XIEP, SWATH technology was applied to do proteome identification, resulting in 163 identified proteins in total (Additional file 1). Among these, 90 proteins belonged to the carbohydrate-active enzymes (CAZymes), including 72 glycoside hydrolases (GHs), 6 carbohydrate esterases (CEs), 11 auxiliary activities (AAs) and 19 carbohydrate-binding modules (CBMs). These enzymes were further annotated to be 11 cellulose-degrading enzymes, 13 xylan-degrading enzymes, 4 chitinases, 4 mannanases, 6 other hemicellulases and 36 hydrolases acting on α/β-1,2/1,3/1,6-glycosidic bonds (Additional file 1). The other identified proteins had functions of expansin, phytase, lipase, hydrophobins (HFBs), small secreted cysteine-rich proteins (SSCRPs), oxidase or unknown functions.

**Fig. 2:**
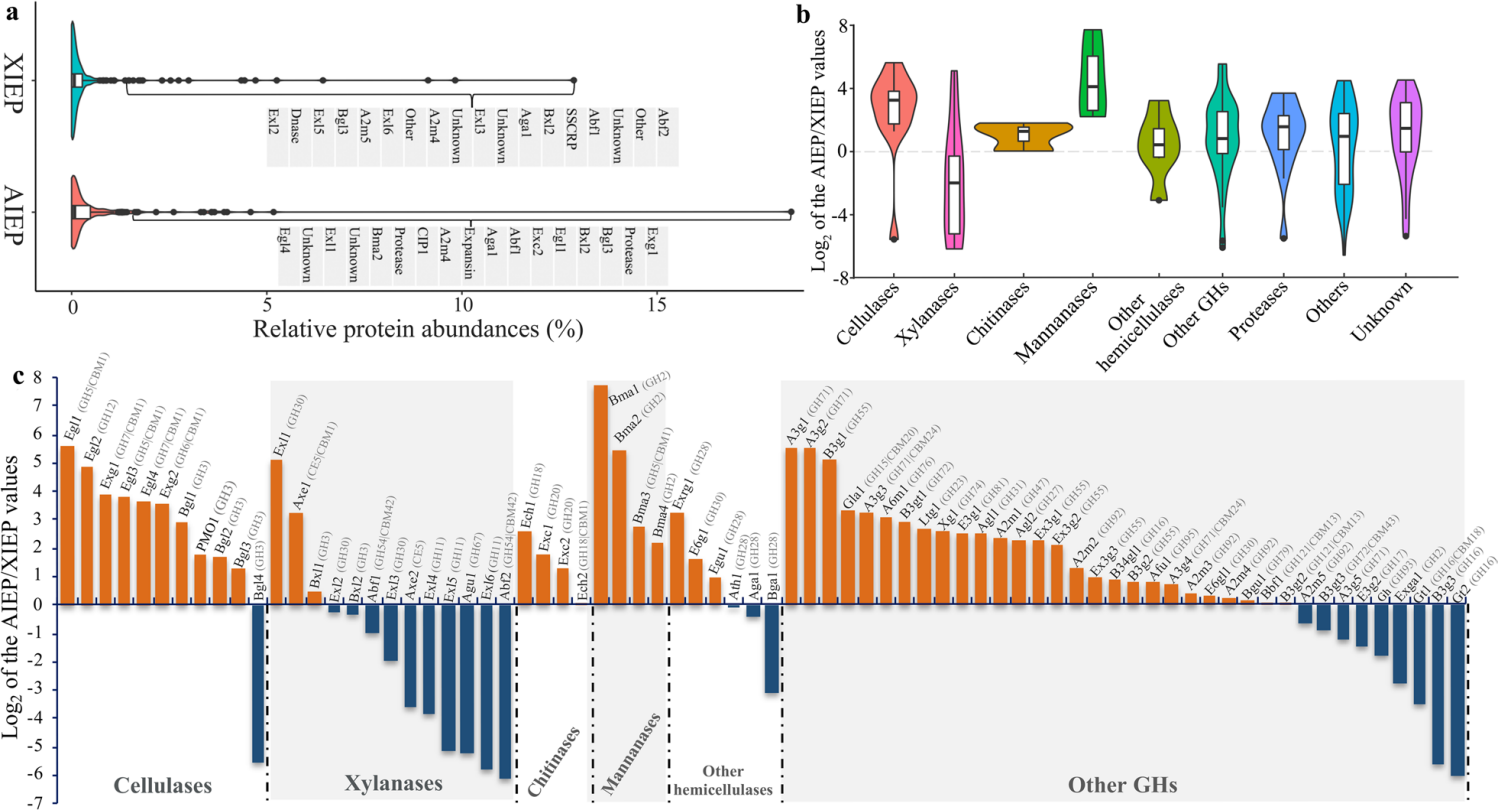
Proteomic analysis of AIEP and XIEP. Extracellular proteins of strain NJAU4742 induced by Avicel or xylan on the fifth day were extracted and identified by the SWATH technique. Violin plot for protein abundances in each sample (AIEP or XIEP) is shown (**A**), and proteins with abundance higher than 1.4% are marked and annotated. In the identified proteins, different groups (cellulases, xylanases, chitinases, mannanases, other hemicellulases, other GHs, proteases, others and unknown) were classified, and the ratio of AIEP/XIEP is shown using the violin plot (**B**). Details, such as the protein annotation, CAZyme families, and AIEP/XIEP values are shown in the histogram for plant biomass-degrading enzymes (**C**). Gene annotations are represented by the corresponding abbreviations. *Egl* endo-β-1,4-glucanase, *Exg* exo-β-1,4-glucanase, *Bgl* β-glucosidase, *PMO* polysaccharide monooxygenase, *Exl* endo-β-1,4-xylanase, *Axe* acetyl xylan esterase, *Bxl* β-xylosidase; Abf, α-L-arabinofuranosidase, *Agu* α-Glucuronidase, *Ech* chitinase, *Exc* Exochitinase, *Bma* β-mannosidase, *Exrg* exo-rhamnogalacturonase, *E6g* endo-β-1,6-galactanase, *Egu* Endo-polygalacturonase, *Ath* α,α-trehalase, *Aga* α-galactosidase, *Bga* β-galactosidase, *A3g* α-1,3-glucanase, *B3g* β-1,3-glucanase, *Gla* glucamylase; A6m, α-1,6-mannanase, *B3gt* β-1,3-glucanosyltransferase, *Ltg* lytic transglycosylase, *Xg* xyloglucanase, *E3g* endo-β-1,3-glucanase; Agl, α-glucosidase; A2m, α-1,2-mannosidase; *Ex3g* exo-β-1,3-glucanase, *B34gl* endo-β-1,3(4)-D-glucosidase, *Afu* α-L-fucosidase, *Bgu* β-Glucuronidase, *Bbf* β-L-arabinobiosidase, *Exga* Exo-β-D-glucosaminidase

Most of the identified proteins in both AIEP and XIEP, accounting for approximately 84.67% and 86.50% of the total proteins, respectively, had low secretion levels (< 1% in abundance) (Additional file 1). This distribution was consistent with the SDS-PAGE profile (Fig. 1b), in which only a few protein bands showed high abundances. Among the 25 and 22 highly abundant proteins (> 1% in abundance) in AIEP and XIEP, 17 and 14 of them were from the GH family, respectively (Fig. 2a), indicating the strong connection between the secreted proteins and carbon hydrolysis. In particular, the highest density band at approximately 63 KDa in AIEP probably corresponded to a CBM1-containing exoglucanase with a protein abundance of 18.44%. Other proteins were more evenly distributed in AIEP than in XIEP, resulting in a higher abundance of extracellular proteins in AIEP (Fig. 2a).

These identified proteins were classified into 9 groups, i.e., cellulases, xylanases, chitinases, mannanases, other hemicellulases, other GHs, proteases, other functional proteins and unknown proteins. Ten of the identified 11 cellulases including 4 endoglucanases, 2 exoglucanases, 4 β-glucosidases and 1 polysaccharide monooxygenase (PMO), were secreted by 2.42 ~ 49.38-fold higher in AIEP than in XIEP (Fig. 2b and c), which is consistent with their measured enzyme activities, showing that Avicel could specifically induce cellulase secretion. The only cellulase with a high abundance in XIEP was a β-glucosidase (Fig. 2c), which also contributes to hemicellulose decomposition as a functional enzyme. Therefore, the specific induction by xylan seems to distinguish it from other β-glucosidases.

The identified 13 xylanases included 3 GH30 endo-β-1,4-xylanases, 3 GH11 endo-β-1,4-xylanases, 2 acetyl xylan esterases, 2 α-L-arabinofuranosidases, 2 β-xylosidases and 1 α-glucuronidase. Among them, 8 proteins, including all of the GH11 endo-β-1,4-xylanases, were highly secreted in XIEP, with 1.97 ~ 70.92-fold higher secretion than in AIEP (Fig. 2b and c). Two β-xylosidases and 1 GH30 endo-β-1,4-xylanase showed no obvious differences between AIEP and XIEP. Two highly secreted xylanases in AIEP were an acetyl xylan esterase and a GH30 endo-β-1,4-xylanase. Obviously, as the most important families (GH10 and GH11) of endo-β-1,4-xylanases in filamentous fungi, all 3 GH11 enzymes assisted by the 5 side-chain-cleaving enzymes were responsible for the high xylanase activities in XIEP. The enrichment of GH30 endo-β-1,4-xylanase in AIEP can explain its unstable and low xylanase activities. In addition, it is possible that the GH30 enzymes have other hydrolysis functions. For example, 2 other identified proteins from the GH30 family were annotated as an endo-1,6-β-D-glucanase and an endo-β-1,6-galactanase (Fig. 2c).

Of the 4 chitinases, 3 of them were enriched 2.42- to 5.88-fold in AIEP, with the remaining chitinase having no difference (Fig. 2b, c), consistent with the detection of high chitinase activities in AIEP when compared to XIEP. The 4 identified mannanases were all enriched in AIEP (Fig. 2b, c). Other enzymes, such as other hemicellulases, other GHs, proteases and other functional proteins and unknown proteins, also had the similar distributions that most of them were enriched in AIEP (58-70%) (Fig. 2b, c).

In general, the proteomic results demonstrated the differences in type and content of extracellular proteins in strain NJAU4742 when induced by two different polysaccharides, Avicel and xylan. Combined with the above results of enzyme activity, we proposed that Avicel and xylan activated the significantly different regulatory networks in strain NJAU4742, which subsequently resulted in the different secretion characteristics of those CAZymes, especially cellulases and xylanases.

### Knocking-out *xyr1* demonstrated the indivisibility of cellulases and xylanases regulatory pathways

**Fig. 3:**
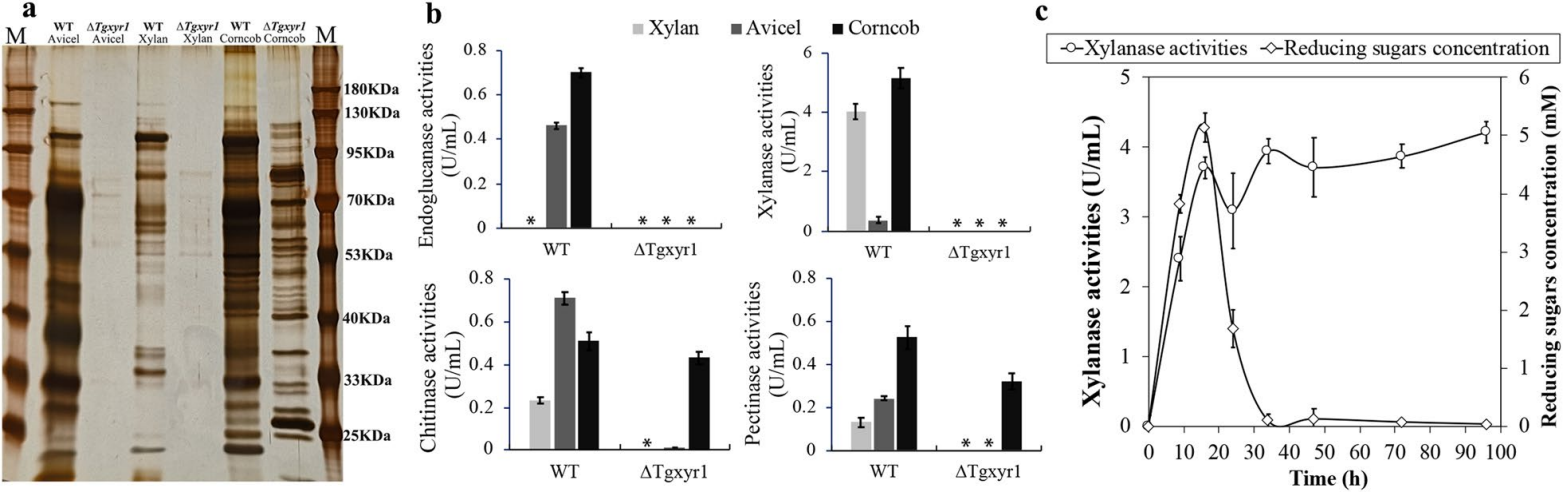
Phenotype analysis of the strain *NJAU4742* and *∆Tgxyr1* mutant. The mycelium of strain NJAU4742 and its *∆Tgxyr1* mutant were cultivated in MM medium using the Avicel, xylan or corncob as the sole carbon source. On the fifth day, extracellular proteins were extracted and detected using SDS-PAGE (**a**), and enzyme activities, including endoglucanase activities, xylanase activities, chitinase activities and pectinase activities, were also determined (**b**). **c** For strain NJAU4742 when induced by xylan, extracellular xylanase activities and reducing sugars concentration were detected in detail. M, Prestained protein ladder; *, no detection values; *WT* wild-type strain NJAU4742. Each detection had three replicates

The above data demonstrated that cellulases and xylanases were obviously controlled by different regulatory pathways in *T. guizhouense* NJAU4742, similar to the regulation model in *Neurospora crassa*, in which xylanases and cellulases are controlled by the transcription factors XLR-1 and CLR-2, respectively [8]. Homologs of XLR-1 and CLR-2 in strain NJAU4742 were identified and designated as TgXyr1 (OPB38038) and TgCLR-2 (OPB38568), respectively, and *∆Tgxyr1* and *∆Tgclr-2* of strain NJAU4742 were thus constructed. Under the induction of 1% (w/v) Avicel or 1% (w/v) xylan, *∆Tgclr-2* had no differences in endoglucanase and xylanase activities when compared with WT NJAU4742 (Additional file 2: Fig S1), and SDS-PAGE also showed similar profiles of extracellular proteins between *∆Tgclr-*2 and WT NJAU4742 (Additional file 2: Fig S1). These results suggested the nonessential role of TgCLR-2 in the regulation of extracellular lignocellulases. However, compared to WT NJAU4742, for *∆Tgxyr1*, only trace levels of extracellular proteins could be detected by SDS-PAGE under the induction of 1% (w/v) Avicel or 1% (w/v) xylan (Fig. 3a), but no any cellulase or xylanase activities (Fig. 3b). These results showed that the regulation of cellulases and xylanases in *T. guizhouense* NJAU4742 were not separated as the case in *N. crassa*, but co-controlled by the transcription factor TgXyr1, which is highly conserved in *Trichoderma species* (Additional file 2: Fig S2).

**Fig. 4:**
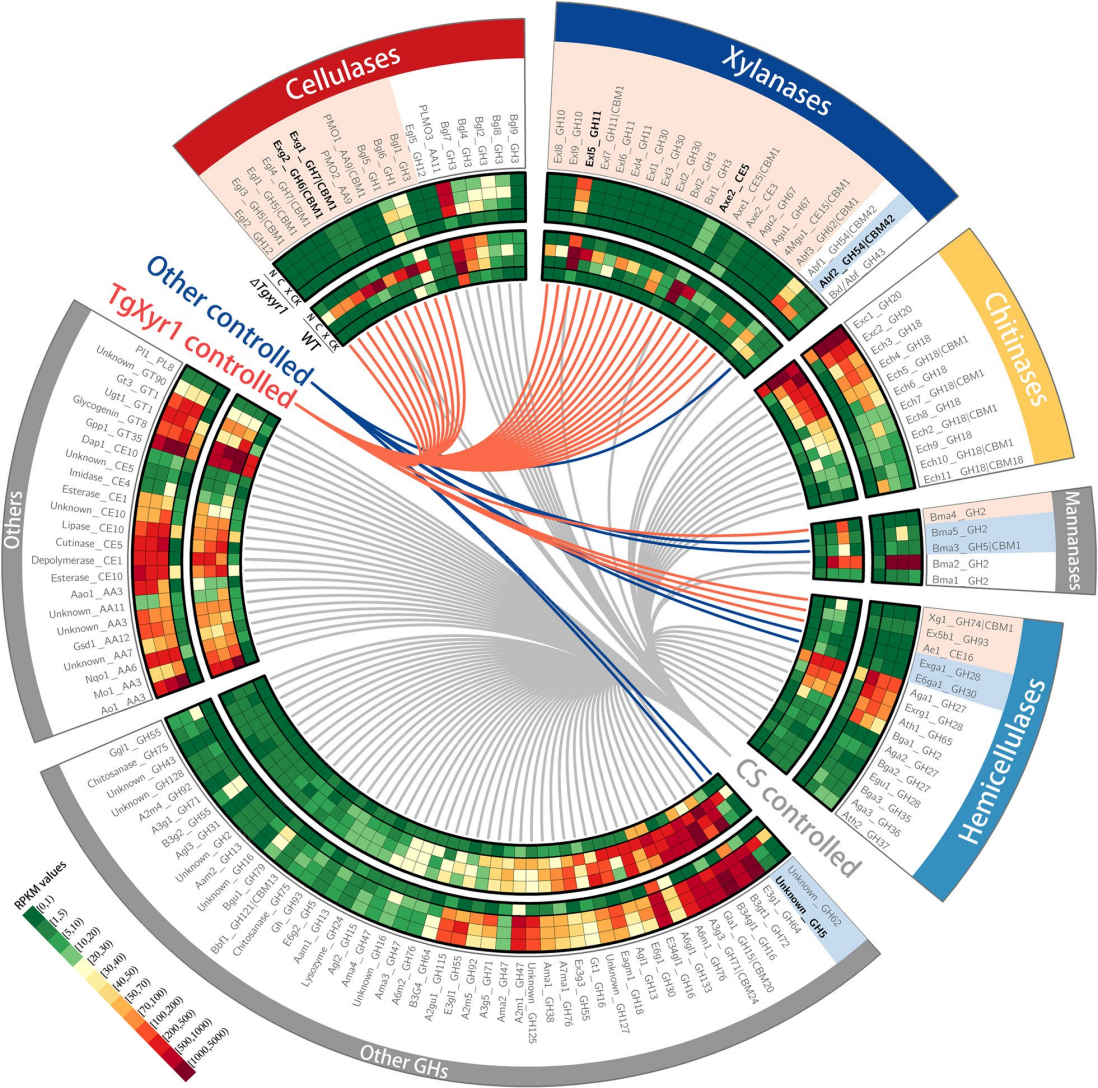
Transcriptome analysis of CAZyme genes in strain NJAU4742 and the ∆*Tgxyr*1 mutant when induced by polysaccharides and carbon starvation. Strain NJAU4742 and the *∆Tgxyr1* mutant were first incubated with glucose and then induced using cellulose, xylan or carbon starvation. Here, we showed the conditions of all polysaccharide/carbon starvation-inducible CAZyme genes in transcriptome data, for which only the highest expression value among the incubation times of 4 h, 24 and 72 h was shown to compare with that value of 0 h for better visualization (see Additional file 3for more information). The first (outermost) circle represents the gene annotation information. The second to the fourth circles represent the carbon starvation-, cellulose- and xylan-induced conditions of the *∆Tgxyr1* mutant, respectively. The fifth circle represents the control at 0 h of the *∆Tgxyr1* mutant. The sixth to eighth circles represent carbon starvation-, cellulose- and xylan-induced conditions of strain NJAU4742, respectively. The last circle represents the control at 0 h of strain NJAU4742. Based on the expression data, genes controlled by TgXyr1, carbon starvation (CS) or other factors were classified and labeled. The color bar indicates the range of RPKM values for each gene. *N* no carbon, *C* cellulose, *X* xylan

Obviously, these results showed a paradox: that enzymatic and proteomic analysis revealed the existence of different regulatory pathways for cellulase and xylanase secretions, but they were controlled by the same regulatory pathway mediated by TgXyr1. To further clarify this complex regulation, we investigated the transcriptomes of WT NJAU4742 and *∆Tgxyr1* when induced by 1% (w/v) Avicel and 1% (w/v) xylan. In total, 476 CAZyme genes were identified and analyzed through the cultivation at different time points of 0 h, 4 h, 24 and 72 h. The results showed that only 33 genes were absolutely controlled by TgXyr1. In WT NJAU4742, these 33 genes were highly induced by Avicel or xylan; however, in *∆Tgxyr1*, most of them had almost no expression (Fig. 4, Additional file 3). These genes included 11 cellulases (4 endoglucanases, 2 exoglucanases, 2 PMOs and 3 β-glucosidases), 18 xylanases (2 GH10 endo-β-1,4-xylanases, 4 GH11 endo-β-1,4-xylanases, 3 GH30 endo-β-1,4-xylanases, 2 β-xylosidases, 3 acetyl xylan esterases, 2 α-glucuronidases, 1 α-L-arabinofuranosidase and 1 4-O-methyl-glucuronoyl methylesterase) and 4 other hemicellulases. qPCR verification showed a similar result, in which all tested 9 cellulose-degrading genes and 9 xylan-degrading genes were highly expressed in strain NJAU4742, however no expression values in *∆Tgxyr1* when induced by Avicel (Additional file 2: Fig S3). All these results clearly showed that strain NJAU4742 used TgXyr1 to absolutely control 6.93% of the CAZyme genes, almost all cellulases and xylanases, to degrade extracellular lignocellulose. In this process, knockout of *Tgxyr1* did not affect the normal transcription of other CAZyme genes. However, the limited carbon source from xylan or Avicel, resulted by the lack of secreted xylanases or cellulases in *∆Tgxyr1*, strongly prevented those CAZymes secretion. When using the natural corncob, which might support some other available carbon sources to *∆Tgxyr1*, there were no detectable cellulase and xylanase activities, as expected; however, significant extracellular protein secretion could be detected (Fig. 3a), and other enzyme activities, such as chitinases and pectinases, were comparable to those of WT NJAU4742 (Fig. 3b). These TgXyr1-related results clearly indicate that cellulases and xylanases are indeed regulated by the same pathway controlled by TgXyr1 in *T. guizhouense* NJAU4742; however, their different regulatory phenotypes that cellulases and xylanases are mostly specifically induced by Avicel and xylan, respectively, remain unclear.

### Transcriptome analysis revealed a multi-level regulation of lignocellulases in *T. guizhouense* NJAU4742

To elucidate the complex regulation of lignocellulases, transcriptome analysis was further performed. Strains NJAU4742 and *∆Tgxyr1* were firstly incubated with 2% (w/v) glucose, and then transferred to the induction of 1% (w/v) xylan or Avicel. Among all CAZyme genes, 41.1% GHs (106/258), 26.9% CE (14/52), 19.3% AA (11/57), 16.7% PL (1/6) and 5.2% GT (5/97) were significantly induced by the polysaccharides (> 4 foldchange of RPKM values and p < 0.01, Additional file 3). These induced genes were divided into cellulases, xylanases, chitinases, mannanases, other hemicellulases, other GHs and others.

For cellulases, there were 8 annotated endoglucanases from the GH5, GH7, GH12 and GH45 families in the genome of strain NJAU4742, 3 of which were not inducible by polysaccharides, but 2 of these non-induced genes had high background expression levels with their RPKM (reads per kilobase per million mapped reads) values of 167 and 676 under glucose cultivation (Additional file 3). The other 5 endoglucanases could be significantly induced by the polysaccharides (> 4 foldchange of RPKM values and p < 0.01) (Fig. 4 and Additional file 3), 4 of which were also identified in the proteomic data (Fig. 2c and Additional file 1). Two annotated exoglucanases from the GH6 and GH7 families were also highly induced and detected in the proteomic data. All 3 annotated PMOs were inducible; however, 1 of them from the AA11 family had a high background expression level (Fig. 4 and Additional file 3), indicating a potential unknown bio-function. For the 17 annotated β-glucosidases, only 8 genes were induced (Fig. 4 and Additional file 3). Consistent with their functions in deconstructing both cellulose and hemicellulose, most induced β-glucosidases (75%) were induced by both Avicel and xylan. However, these dynamic transcriptome data clearly showed that nearly all main cellulases, including 4 endoglucanases, 2 exoglucanases, 2 PMOs and 2 β-glucosidases, were significantly induced only by Avicel (> 4 foldchange of RPKM values and p < 0.01, Fig. 4 and Additional file 3), supporting the fact that cellulose specifically induced cellulases in strain NJAU4742.

80% of the annotated xylanases, including 9 endoxylanases (2 GH10, 4 GH11 and 3 GH30 families), 5 acetyl xylan esterases, 6 β-xylosidases, 2 α-L-arabinofuranosidases and 1 4-O-methyl-glucuronoyl methylesterase were induced by the polysaccharides (Fig. 4 and Additional file 3). As the major endoxylanases of filamentous fungi, the 2 GH10 endoxylanases had no significant expression under the xylan induction but were highly induced by Avicel (> 4 foldchange of RPKM values and p < 0.01, Fig. 4 and Additional file 3). This result indicates the special induction of GH10 endoxylanases by cellulose but not xylan in strain NJAU4742. However, the Avicel specifically highly indued GH10 endoxylanases were not detected in the proteomic identification of AIEP (Fig. 2c and Additional file 2), and also conflicted with the low extracellular xylanase activities of AIEP (Fig. 1i). Similarly, all 4 GH11 endoxylanases were also highly induced by Avicel with the level significantly higher than or similar to those by xylan (Fig. 4 and Additional file 3). This better induction by Avicel also conversely leaded to much lower extracellular protein abundances and enzyme activities of GH11 endoxylanases in AIEP when compared to XIEP (Fig. 2c and Additional file 1). qPCR verification supported the Avicel-specific induction of GH10 xylanase genes and comparable induction of GH11 xylanase genes with xylan (Additional file 2: Fig S3). Thus, the results seem to support a function of the post-transcriptional inhibition (PTI) existed on xylanase secretion in strain NJAU4742. And, this deduced PTI is removable, for example, the 2 GH10 endoxylanases were exactly identified in extracellular proteins at the fourth incubation day when strain NJAU4742 was fermented with rice straw in our previous study [23]. Meanwhile, extracellular enzymes induced by the corncob in this study also showed high xylanase and cellulase activities together (Fig. 3b). Certainly, the existence or not of the PTI on xylanase secretion should be discussed in detail.

The annotated chitinases include 21 GH18 endochitinases and 2 GH20 exochitinases, of which 10 endochitinase genes and 2 exochitinase genes were significantly induced by the polysaccharides (> 4 foldchange of RPKM values and p < 0.01, Fig. 4 and Additional file 3). Exochitinase 1 (Exc1) had a high background expression level (RPKM = 364 under glucose cultivation). The non-induced endochitinase (OPB36499) also had a high background expression value of RPKM 112 under glucose cultivation. For the other enzymes, 71.4% of the remaining hemicellulases were induced by polysaccharides, showing the importance of hemicellulases in lignocellulose deconstruction. Among the other 161 GHs, 31.1% were induced by polysaccharides, mainly the α/β-1,2/3/6-glycoside hydrolases.

### Carbon starvation-induced transcriptome helped to understand the whole regulatory process of carbon utilization in strain NJAU4742

To further understand the mechanism of CAZymes regulation, we also performed the transcriptome analysis under carbon starvation (CS) for both strains NJAU4742 and *∆Tgxyr1* at the induction times of 0 h, 4 h, 24 and 72 h, respectively. The results showed that CS totally induced 109 CAZyme genes, which were completely included in the 143 polysaccharide-induced CAZyme genes (Fig. 4 and Additional file 3). Therefore, we classified these genes into 5 different groups as their regulation (Fig. 4). The first group was defined as CS specifically controlled genes, which contained 103 genes that were induced but no significant difference in their induction levels between polysaccharides and carbon starvation. Genes only induced by polysaccharides, were defined as TgXyr1 specifically controlled genes, of which *TgXyr1* knockout completely blocked their expressions (29 genes, the second group), or the other factors specifically controlled genes (4 genes, the third group). There were 5 genes that were highly induced by the polysaccharides and slightly induced by the CS, TgXyr1 knockout would decrease the original high polysaccharides-induction levels to the low CS-induction levels in 4 genes of them. These 4 genes were thus defined as TgXyr1 and CS co-controlled genes (the fourth group), and the left gene was co-controlled by the other factors and CS (the fifth group). From this information, we can speculate that when carbon source is lacking in the environment, strain NJAU4742 tended to activate many different glycoside hydrolases, thereby fully covering the environmental carbon spectrum to obtain the sufficient carbon source and energy for survival. However, to obtain carbon source from lignocellulose, strain NJAU4742 only slightly activated 4 lignocellulose-degrading genes (2 exoglucanases, 1 GH11 endoxylanase and 1 acetyl xylan esterase) before largely secreting all 33 TgXyr1-controlled lignocellulases if confirming the existence of lignocellulose components. This strategy for lignocellulose utilization was obviously more economical.

## Discussion

Filamentous fungi secrete a variety of lignocellulosic enzymes through a complex regulatory system, in which the regulatory pathways of different enzymes have strong interactions with each other, and thus need to be understood. In this study, *T. guizhouense* NJAU4742 was induced by the polysaccharides of xylan and cellulose, and it responded by secreting different extracellular proteins, AIEP and XIEP. As the main polysaccharide-degrading enzymes, cellulase activities were specifically detected in AIEP, while, xylanase activities were mainly detected in XIEP. Proteomic analysis also demonstrated this phenomenon, i.e., the main cellulases (Egls, Exgs, PMOs and Bgls) were enriched in AIEP, while the main xylanases (GH11 Exls, Afbs, Agus and Axes) were enriched in XIEP. Therefore, we speculated that cellulases and xylanases were independently regulated in strain NJAU4742. Similar results were also reported in *N. crassa*, in which xylan could not induce any cellulase activities, and the corresponding mechanism showed that cellulases were controlled by the CLR-1/CLR-2-mediated pathway, while xylanases were controlled by Xyr1 [9]. However, *∆Tgxyr1* and *∆Tgclr-2* in the strain NJAU4742 background demonstrated that TgCLR-2 did not have a role in cellulases regulation, and TgXyr1 deletion disrupted both cellulase and xylanase secretions under all tested conditions. Transcriptome analysis indicated that nearly all cellulase and xylanase genes were absolutely dependent on TgXyr1 for expression. These results, however, indicated that both cellulases and xylanases were under the control of the same TgXyr1-mediated regulatory pathway. Thus, the above two contradictory conclusions prompted us to think about how cellulases and xylanases could specifically respond to different extracellular polysaccharides while simultaneously being controlled by the same Xyr1-mediated regulatory pathway in *T. guizhouense* NJAU4742.

Transcriptome analysis gave the result that cellulase genes were specifically induced by cellulose but not xylan. However, TgXyr1 was strongly induced by both cellulose and xylan in strain NJAU4742 (Additional file 3), but the highly expressed TgXyr1 did not activate cellulase genes it controlled under xylan induction. Two possible hypotheses were thus proposed for this phenomenon: (1) Similar to the function of CLR-3 in *N. crassa* [9], a specific repressor may exist by directly inhibiting cellulase gene expression, and this repression can only be removed by cellulose induction. This putative repressor will not be the CLR-3 homolog in strain NJAU4742 due to its function by repressing CLR-1/CLR-2 pathway, which, however, does not work in strain NJAU4742. (2) In addition to the TgXyr1, there may be another specific activator of cellulase genes, which can be specifically induced by cellulose and is as indispensable as TgXyr1 for cellulase gene expression. In *Trichoderma reesei*, some similar cellulase activators have been reported, such as RXE1 and ACE3. RXE1 functions by activating Xyr1 expression, and it was thus excluded from our speculation [19]. ACE3 could specifically bind to most cellulase gene promoters, in which ACE3 interacts with the Xyr1 regulator, and ACE3 deletion stops cellulase secretion [15, 24]. This information is very consistent with our speculation; however, a homolog of ACE3 (OPB36832), similar to TgXyr1, was significantly induced by both cellulose and xylan in strain NJAU4742 (> 4 foldchange of RPKM values and p < 0.01, Additional file 3). In addition, ACE3 was also reported to bind Xyr1 promotor and thus affect xylanase gene expression indirectly [15]. Ultimately, this analysis reflected the possibility of an unknown cellulase-specific regulator, which is expected to be the necessary but insufficient for cellulase secretion by strain NJAU4742.

For xylanase activity, the PTI function was deduced mainly for the difference between xylanase transcription and secretion. Considering the detection time of transcriptome (0–72 h) and proteome (5th d), the possible reason could be concluded that GH10 endoxylanases might be degraded or inhibited in time region from 72 h to 5 d. In *T. reesei*, inhibition of xylanase transcription really happened during the later stages of fermentation [14]. However, a logical error existed that the highly indued GH10 endoxylanases should be reflected as extracellular xylanase activity at least one certain stage of the fermentation process, which, however, is indeed that Avicel-induced activities of xylanases maintain a quite stable and low state during the fermentation of 7 days in strain NJAU4742 (Fig. 1i). Of course, the easy degradation of xylan released much more soluble sugars, which possibly activated CCR during the process, and thus might contribute to the low protein secretion in XIEP. Nonetheless, CCR effect theoretically limited the further increase of xylanase secretion in XIEP, so it did not affect the conclusion that xylan can specifically induce xylanase secretion. The whole phenomenon was difficult to explain, somewhat similar to that of *∆Tgxyr1*, in which the uncontrolled genes, including chitinases, pectinases etc., were highly induced by Avicel or xylan, however there were no obvious extracellular enzyme activities. The lack of carbon source explained the phenomenon of *∆Tgxyr1*, but in strain NJAU4742, Avicel degradation could provide enough soluble sugars for large secretion of extracellular proteins (Fig. 1a). Therefore, the phenomenon of high induction but quit low secretion of xylanase under cellulose induction was not caused by lack of carbon source. On the other hand, the problem might also come from the calculation of xylanase activities, which was actually not normalized to strain biomass. Thus, higher strain growth fermented by xylan might interfere the comparison of secreted xylanases between AIEP and XIEP. For verification, xylan fermentation of strain NJAU4742 was further performed and showed that xylanase activity was produced rapidly (60% at 9 h and nearly 100% at 16 h after induction) (Fig. 3c). The mycelia amount at 9–16 h in XIEP should be comparable to those of AIEP or even to those of AIEP at the fifth induction day, however, xylanase activities still had a big difference (Fig. 1i). In addition, we also tried to detect the strain biomass when induced by xylan or Avicel as accurate as possible (see Methods). They both reached the similar maximum level after the 3th incubation day, even though the biomass in xylan culture increased faster than that in Avicel culture in the early stage (< 48 h) (Additional file 2: Fig S4). Thus, biomass difference actually did not affect the conclusions judged after the 3th incubation day, such as those for the enzyme activities, proteomic analysis and transcriptome analysis. All in all, the analysis strongly suggests the existence of a post-transcriptional inhibition (**PTI**) on xylanases when strain NJAU4742 induced by Avicel. PTI inhibited xylanase translation or transportation and thus produced much lower extracellular activities in AIEP. In addition, the homologs of ACE1 and ACE2 regulators in *T. reesei* were also found in strain NJAU4742, and designated as TgACE1 (OPB46882) and TgACE2 (OPB44681). Transcriptome analysis of *∆Tgace1* and *∆Tgace2* mutants induced by Avicel/xylan did not clarify their functions, however, both CAZyme expression profiles showed similar to that of strain NJAU4742 ( Additional file 3), and thus completely supported the conclusion that Avicel almost specifically induced GH10 xylanases, and also largely induced GH11 xylanases. Therefore, PTI hypothesis seems to be reliable, however, still need much more experiments for verification.

When it comes to regulation of xylanases, both PTI and transcriptional activation should co-exist. As the main xylanases, endoxylanase can be mainly classified into the GH10 and GH11 families. The GH10 endoxylanases have greater catalytic versatility than GH11 and have also been reported to have excellent cellulase activity [25, 26]. The findings here revealed that their different substrate activities have already allowed GH10 endoxylanases to form a regulatory pathway independent from the other major xylanases. On the basis of these facts, we can propose that the transcriptional activation and PTI of xylanase genes are relatively independent. Similar to cellulase genes, an unknown specific repressor or activator should exist to control the expression of the 2 GH10 endoxylanases.

In addition, we also investigated the effect of carbon starvation on CAZymes induction. Surprisingly, all carbon starvation-induced CAZyme genes were among the polysaccharide-induced CAZyme genes, accounting for approximately 76.2% of the latter. Even more surprisingly, the remaining 23.8% of polysaccharide-induced genes were almost exclusively those genes controlled by TgXyr1. Only a few TgXyr1-controlled genes had the slight induction levels during the starvation condition. From these results, it could be concluded that strain NJAU4742 tended to express many hydrolases, thereby fully covering the environmental carbon spectrum to obtain sufficient carbon source and energy for survival. This large-scale expression of degrading enzymes should be regulated systematically. For example, the highly induced genes only produced trace extracellular proteins, which indicated the existence of protein secretion inhibition in carbon starvation to balance the economic benefits of substance release and acquisition. In addition, among all of the TgXyr1-controlled genes, only 4 genes (2 exoglucanases, 1 GH11 endoxylanase and 1 acetyl xylan esterase) were slightly induced by carbon starvation, which revealed that strain NJAU4742 only selected to slightly secret several scouting enzymes, before sending a full set of specific enzymes (TgXyr1-controlled degrading enzymes) if any polysaccharides existed in the environment, to avoid wasting resources. This phenomenon could be also observed in *Aspergillus niger* and *N. crassa* [27]. For the same reason, the role of scouting enzymes may also be played by some of the other CS-induced CAZyme genes, and further activations may be blocked if there are no corresponding substrates.

TgXyr1 could also be highly induced by carbon starvation (Additional file 3), however, did not initiate the expression of its controlled genes under this condition. In *T. reesei*, Xyr1 mutant Xyr1 A824V, was reported to secret cellulases when cultivated by glucose [28], and this phenotype is commonly applied in other filamentous fungi to enhance the degradability of lignocellulose [29, 30]. However, the related mechanism has not yet been revealed. We thus hypothesize that, due to the lack of interaction with other activators, highly induced TgXyr1 cannot turn on the transcription of degrading enzymes under CS condition in strain NJAU4742. As recently reported, the TrCYC8/TUP1 complex functions as a novel coactivator for Xyr1 in the promoter region of degrading enzymes in *T. reesei* [31]. Meanwhile, the negative effect may also be involved in the repression of TgXyr1-controlled genes under CS condition, for example, the hyperosmotic-response (OS) pathway reported in *N. crassa* [9]. Blocking the OS pathway could promote cellulase secretion under CS condition in *N. crassa*. These global mechanisms of positive and negative regulations should coexist and function together. In fact, lignocellulase regulation is much more complex in strain NJAU4742, for example, the TgXyr1-controlled enzymes were all inhibited in CS condition, but how could be selectively inhibited by different polysaccharides? Specific repressors and activators probably existed, and additional research is necessary to identify them.

## Conclusions

**Fig. 5:**
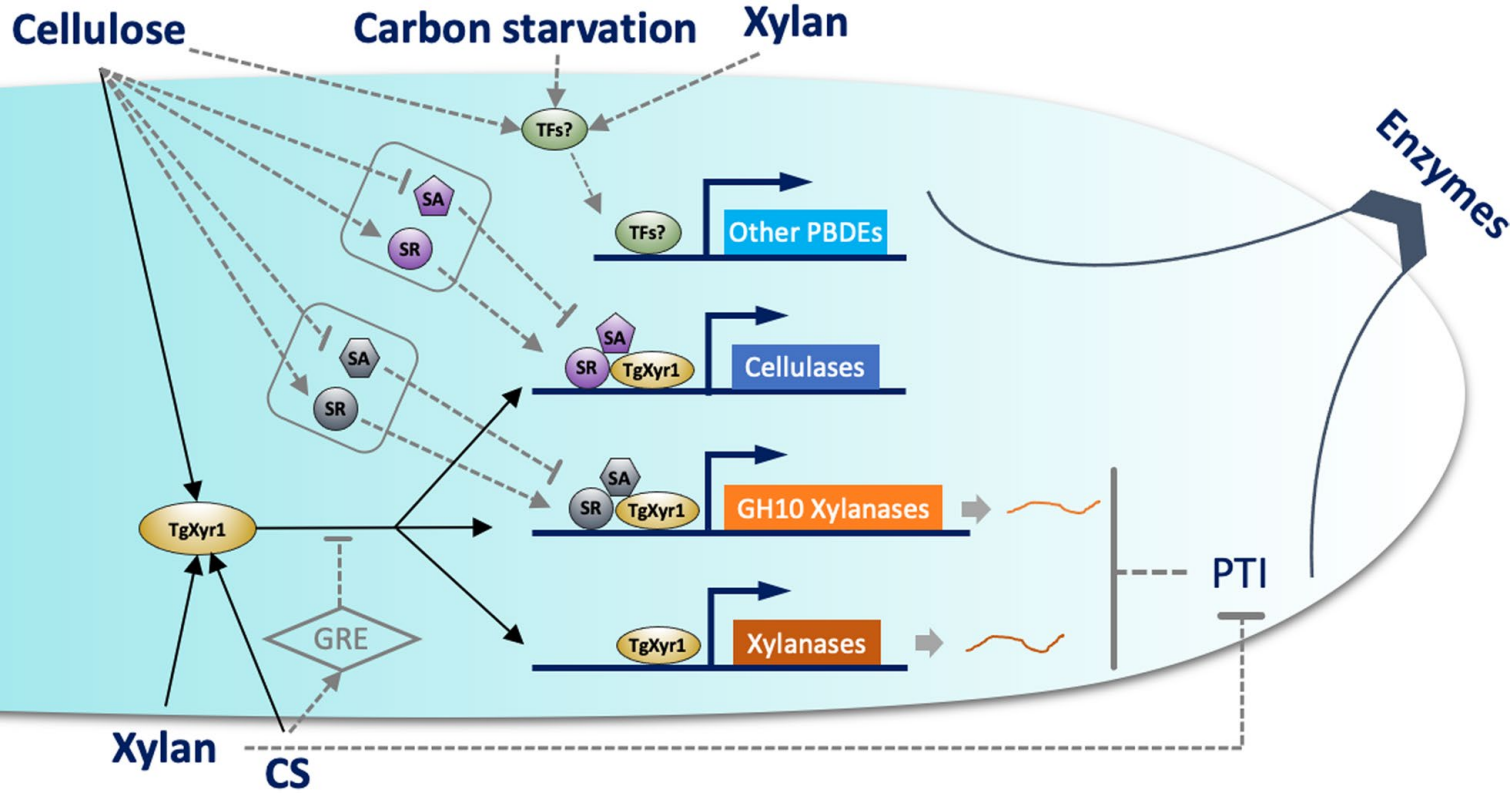
Schematic diagram of the regulatory mechanism of PBDEs in strain NJAU4742. Cellulases and xylanases were absolutely controlled by TgXyr1; however, they could also respond to different polysaccharides, among which cellulases and GH10 xylanases could be specifically induced by cellulose, while the other main xylanases could be induced by both cellulose and xylan. The specific activator (SA) or specific repressor (SR) is suspected to be responsible for the specific induction of cellulases and GH10 xylanases by cellulose. The PTI mechanism was employed to repress all xylanase genes when induced by cellulose to reflect the phenotype in which xylan specifically induced xylanase secretion. In addition, carbon starvation, together with cellulose and xylan, could induce other transcription factors, which initiate the expression of other plant biomass-degrading enzymes (PBDEs), except cellulases and xylanases. Some unknown global repression effects (GREs) also existed to repress the expression of cellulases and xylanases when induced by carbon starvation. The gray pathways show the results of this study

This study used an efficient biomass-degrading strain, *Trichoderma guizhouense* NJAU4742, as an object to compare and analyze the extracellular enzymes and their corresponding transcriptional responses when induced by different polysaccharides or during carbon starvation. From these results, on the basis of the current knowledge from the different filamentous fungi, we provide a preliminary understanding of the regulatory mechanisms of lignocellulosic enzymes, which is shown in Fig. 5. These novel propositions will provide research directions for further analysis of the complex carbon utilization system in filamentous fungi, and also contribute to the artificial modification of high-efficiency degrading strain for plant biomass.

## Methods

### Strains and culture conditions

*Trichoderma guizhouense* NJAU4742 (genomic sequence accession no. in the NCBI database: LVVK00000000.1) was isolated from a mature compost sample in Nanjing of China. Derived mutants (*∆Tgxyr1*, *∆Tgclr-2*, *∆Tgace1* and *∆Tgace2*) were constructed in this study. The strains were cultivated in minimal medium (MM, 5 g·L^− 1^ (NH_4_)_2_SO_4_, 15 g·L^− 1^ KH_2_PO_4_, 0.6 g·L^− 1^ MgSO_4_·7H_2_O, 1 g·L^− 1^ CaCl_2_, 1.6 mg·L^− 1^ MnSO_4_·H_2_O, 1.4 mg·L^− 1^ ZnSO_4_·7H_2_O, 5 mg·L^− 1^ FeSO_4_·7H_2_O, 2 mg·L^− 1^ CoCl_2_·6H_2_O) with different carbon sources (glucose, xylan, Avicel or corncob). Briefly, 10^7^ spores from strain NJAU4742 or its mutants were inoculated into 200 mL liquid MM supplemented with 2% (w/v) glucose at 28 ℃ and 150 rpm. After 48 h, the mycelia were harvested and washed thoroughly with sterile water, and then transferred into fresh 200 mL liquid MM with the sole carbon source of 1% (w/v) xylan (sugarcane bagasse xylan, Yuanye, China), 1% (w/v) cellulose (Avicel PH101, Sigma, China), 1% (w/v) corncob or no carbon source. At each sampling time (0 h, 4 h, 24 and 72 h for transcriptome analysis; 0 h, 24 h, 48 h, 72 h, 96 h, 120 h, 144 and 168 h for enzyme activity assays), 10 mL fermentation broth was sampled to collect the mycelia by filtering through two layers of gauze, and culture supernatant through a centrifugation at 6000 rpm for 5 min followed by a filtration using a 0.22 μm sterile membrane. All collected samples were immediately stored at -80 ℃ for further analysis. The corncob, as an available natural material of polysaccharides mixture, contains cellulose (27.71%), hemicelluloses (38.78%) and lignin (9.4%) [32], and was thus ground and used (65 mesh) in this study. Three biological repeats existed for each sampling point.

## Mutant construction

Gene editing was performed using hygromycin B (HygB) for screening according to our previous method [22]. Briefly, the donor DNAs of *Tgxyr1*, *Tgclr-2*, *Tgace1* and *Tgace2* were constructed in the form of UF (upstream fragment of the target gene) + *hph*-EC (*hph* gene expression cassette) + DF (downstream fragment of the target gene) by fusion PCR using CloneAmp HiFi PCR Premix (TAKARA, Japan). Protoplasts of strain NJAU4742 were then prepared using 7.5 mg·mL^− 1^ lysing enzyme (Sigma: L1412, China) dissolved in solution A (1.2 M sorbitol, 0.1 M KH_2_PO_4_, pH 5.6). Next, 10 µl DNA (≥ 1 µg) was transformed into 200 µL protoplasts (≥ 1 × 10^8^ protoplasts/mL) by the PEG-CaCl_2_ method [22]. Protoplast regeneration was achieved by spreading the transformation mixture evenly onto 1 M sucrose-containing PDA plates for 24 h at 28 ℃, which were then covered with 0.2 mg·mL^− 1^ HygB-containing PDA medium. After several days (2–4 d), the colonies growing normally on the HygB-containing PDA medium were transferred into fresh HygB-containing PDA plates and verified by PCR. All primers used are showed in Additional file 2: Table S1.

## Enzyme activity assays

The culture supernatants from the liquid fermentation of the *Trichoderma* strains induced by different carbon sources were directly used in this experiment. Endoglucanase, exoglucanase, filter paper enzyme, xylanase, chitinase, pectinase and mannanase activities were measured using carboxymethyl cellulose sodium (CMC-Na) (Sinopharm Chemical Reagent Co., China), *p*-nitrophenyl-β-D-cellobiose (*p*NPC) (Sigma, USA), filter paper (Whatman NO0.1, UK), sugarcane bagasse xylan (Yuanye, China), chitin (Yuanye, China), pectin from citrus peel (Sigma, USA) and locust bean gum from *Ceratonia siliqua* seeds (Aladdin, China) as the substrates, respectively, according to the methods described previously [33]. One unit of enzyme activity was defined as the amount of enzyme required to release 1 µmol of reducing sugars or *p*NP from the substrate in 1 min. All of the enzyme activities are reported as the means of at least three replicates.

## Strain biomass detection

Equal amounts of mycelia were transferred into fresh 200 mL liquid MM with the sole carbon source of 1% (w/v) xylan or 1% (w/v) Avicel. At each sampling time (0 h, 24 h, 48 h, 72 h, 96 h, 120 h, 144 and 168 h), 10 mL fermentation broth was sampled. Mycelia in the sample was firstly filtrated with Miracloth (Calbiochem, USA), and then washed by gentle shaking using the sterile water in a 15 mL tube. The above steps were repeated for 3–5 times to remove xylan or Avicel particles as much as possible. When observing the enough clear suspension, the mycelia was blotted dry through filter paper after a final filtration, and followed by weighting. Each sample had three replicates.

## Protein extraction, SDS-PAGE and SWATH analysis

The protein concentration was determined using a Micro BCA protein assay kit (Beyotime, China). Sodium dodecyl sulphate-polyacrylamide gel electrophoresis (SDS-PAGE) was performed according to Laemmli [6] on a 10% (w/v) polyacrylamide gel with a PageRuler Prestained Protein Ladder (Fermentas, China) using a Mini-PROTEAN Tetra Cell System (Bio-Rad, China). The liquid fermentation broth at the fifth day was collected by centrifugation at 10,000 rpm for 10 min and then filtered through a 0.22 μm sterile membrane. Protein samples of Avicel- and xylan-induced extracellular proteins in strain NJAU4742 were then concentrated by lyophilization, and commissioned to a specialized company (GeneCreate, Wuhan, China) for analysis of the sequential window acquisition of all theoretical fragment ions (SWATH analysis). The detailed steps were described in our previous research [23]. Briefly, Equal amount of protein from each sample (100 µg) were used for tryptic digestion. Then, peptides were desalted using C18 columns, and dried with Vacuum concentration meter. LC-MS/MS analysis was performed on an AB SCIEX nanoLC-MS/MS (Triple TOF 5600 plus) system in two phases: data-dependent acquisition (DDA) was followed by SWATH acquisition, where all samples were firstly mixed and detected in DDA, and the resulting data were used as a library for analysis of each sample by SWATH. Spectral library generation and SWATH data processing were performed using the Peakview version 2.2 software. Fragment ion areas that belonged to one peptide were added to obtain a peptide’s abundance, and the total abundance of peptides for a given protein was determined to obtain the protein’s abundance. To eliminate the random errors and sample bias, all the data among samples were normalized using the median normalization method [34], and the mProphet algorithm was used both for sample normalization and for assessing the data confidence [35]. Each sample had three biological replicates.

## qPCR analysis

Strains NJAU4742 and its mutants *∆Tgxyr1* were firstly incubated with 2% (w/v) glucose, and then transferred to the induction of 1% (w/v) xylan or Avicel. After 24 and 72 h incubation, mycelium was harvested and frozen immediately in liquid nitrogen. Total RNA was extracted using the RNeasy Plant Mini Kit (Qiagen, Germany) combined with the RNase-Free DNase set (Qiagen, Germany), and ~ 1 µg of total RNA was used for cDNA synthesis with HiScript® II Q RT SuperMix for qPCR (+ gDNA wiper) (Vazyme). qPCR was performed with the ChamQ SYBR qPCR Master Mix (Vazyme) and the expression level of each gene was normalized to the translation elongation factor 1 alpha (*tef1*) gene. Primers used in this study are listed in Additional file 2: Table S2.

## RNA sequencing and transcript abundance

The sampled mycelia were stored at -80 ℃, and then disrupted by grinding in liquid nitrogen, and subsequently used to extract total RNAs using the RNeasy Plant Mini Kit (Qiagen, Germany) combined with the RNase-Free DNase set (Qiagen, Germany). RNA degradation and contamination were monitored on 1% agarose gels. Transcriptome data were obtained from an Illumina HiSeq™ PE150 (Novogene, China) as described previously [36] with three biological replicates for each sample. The raw sequences generated were filtered for quality using FASTX-Toolkit (http://hannonlab.cshl.edu/fastx_toolkit/index.html). All of the high-quality sequences were aligned against the NJAU4742 genome with the spliced aligner Bowtie2 integrated in TopHat2 [37]. Cuffnorm and Cuffdiff were used for normalization and differential expression analyses. Expression differences characterized by |log2 of foldchanges| ≥ 2, a *p*-value ≤ 0.01 and FPKM values > 2.0 were considered significant. The RNA-seq data reported in this study have been deposited in the NCBI Gene Expression Omnibus and are accessible through GEO Series accession number GSE176374.

## Bioinformatic analysis

For strain NJAU4742, a gene annotation table was downloaded from the NCBI genome database, and then all CAZyme genes were verified and updated manually using the Blastp program on the NCBI non-redundant protein database. CAZymes annotation was performed using the hmmscan tool from HMMER 3.1 software to search the dbCAN database v4 [38], and the output was processed using the script of hmmscan-parser.sh (https://github.com/carden24/Bioinformatics_scripts/blob/master/hmmscan-parser.sh). Signal peptide was predicted using SignalP 5.0 software (https://services.healthtech.dtu.dk/). Violin plot was carried out using the R package vioplot. Sequence alignments were performed with ClustalW, and the neighbor-joining tree was generated with MEGA 5.1 software. Different enzyme groups, such as cellulases, xylanases and chitinases, were classified by considering gene annotations, CAZyme families and degradational mechanisms together. CIRCOS v0.67-7 software (https://www.circos.ca) was used for transcriptional data visualization.

## Supplementary Information

**Additional file 1. **The processed dataset of SWATH results

**Additional file 2. **The phenotype of *∆Tgclr-2*, phylogenetic tree of TgXyr1, qPCR verification, growth curve of strain NJAU4742 and all used primers in this study

**Additional file 3.** The processed dataset of transcriptomic results
