# Supplementary material for: Exploring the multi-level regulation of lignocellulases in the filamentous fungus Trichoderma guizhouense NJAU4742 from an omics perspective
Source: Microb Cell Fact. 2022 Jul 16;21:144. doi: 10.1186/s12934-022-01869-3 (PMC9288086; doi:10.1186/s12934-022-01869-3)
Supplement: Supplementary file 2 — Additional file 2. The phenotype of ∆Tgclr-2, phylogenetic tree of TgXyr1, qPCR verification, growth curve of strain NJAU4742 and all used primers in this study [file 12934_2022_1869_MOESM2_ESM.docx]

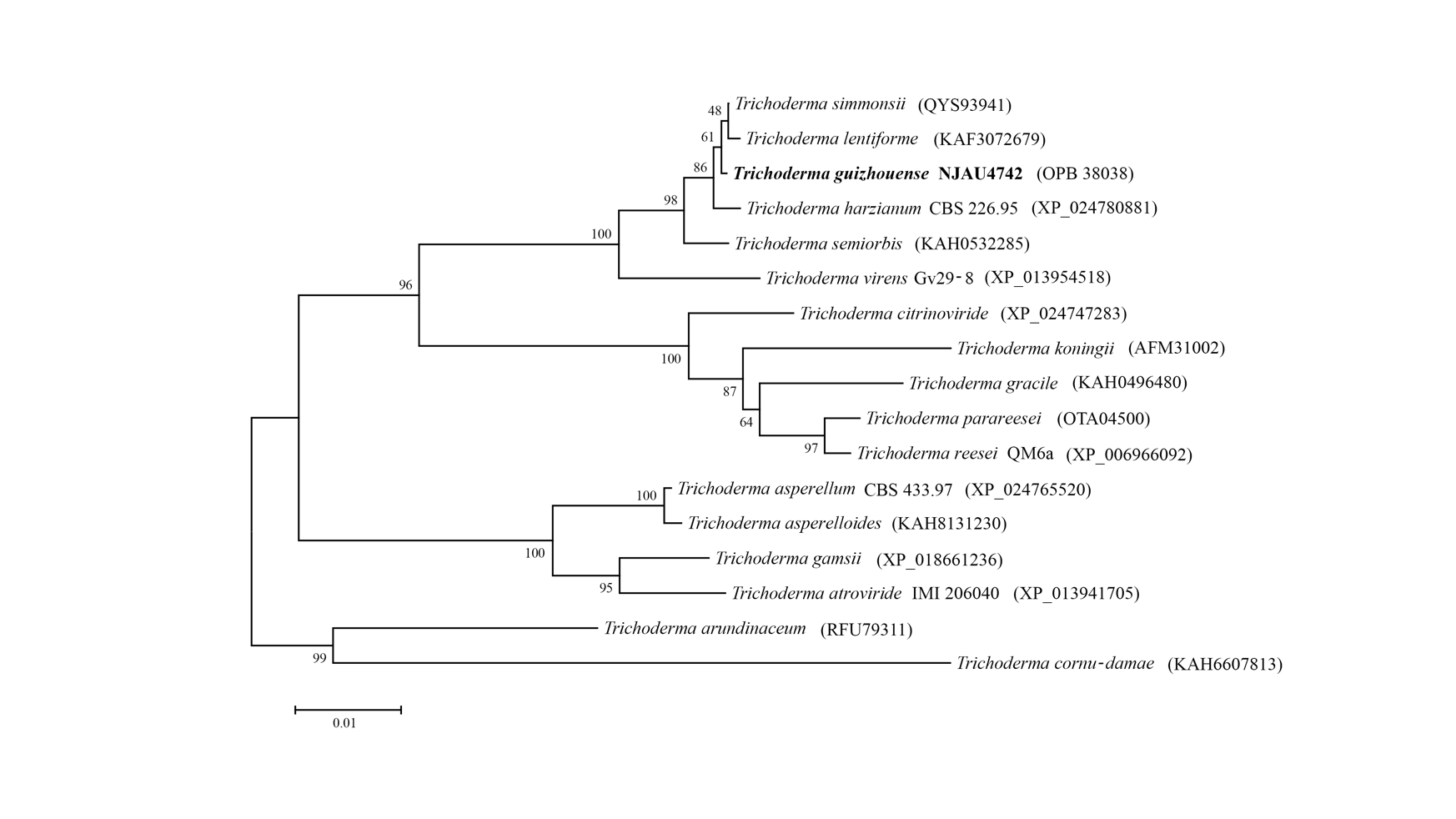
**
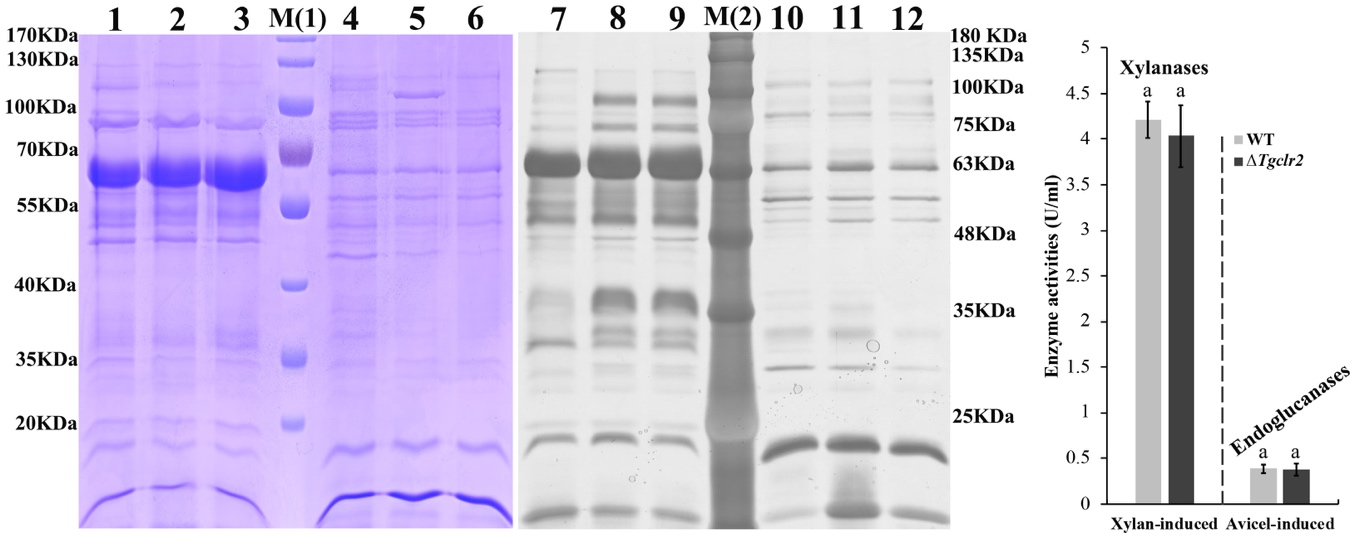
Fig S1. The phenotype of *∆Tgclr2* mutant.** WT strain NJAU4742 and *∆Tgclr2* mutant were incubated with 1% (w/v) Avicel or 1% (w/v) xylan for 5 days. Extracellular proteins on the 5^th^ day were extracted and detected by SDS-PAGE. Line 1-3 and line 4-6 stand for the three biological replicates of Avicel- and Xylan-induced extracellular proteins from *∆Tgclr2* mutant, respectively. Line 7-9 and line 10-12 stand for the three biological replicates of Avicel- and Xylan-induced extracellular proteins from WT strain NJAU4742, respectively. M(1) and M(2) are two different protein markers. Xylanase activities and endoglucanase activities were also detected and shown here.

**Fig S2. The phylogenetic tree of TgXyr1 in *Trichoderma* species.** The homologs of TgXyr1 in *Trichoderma* species were searched and downloaded using TgXyr1 protein sequence blasted to NCBI nr-database. Sequence alignments were performed with ClustalW, and the neighbor-joining tree was generated with a bootstrapping analysis of 1000 replicates in MEGA 5.1 software. All accession numbers of TgXyr1 homologs were shown in the figure.


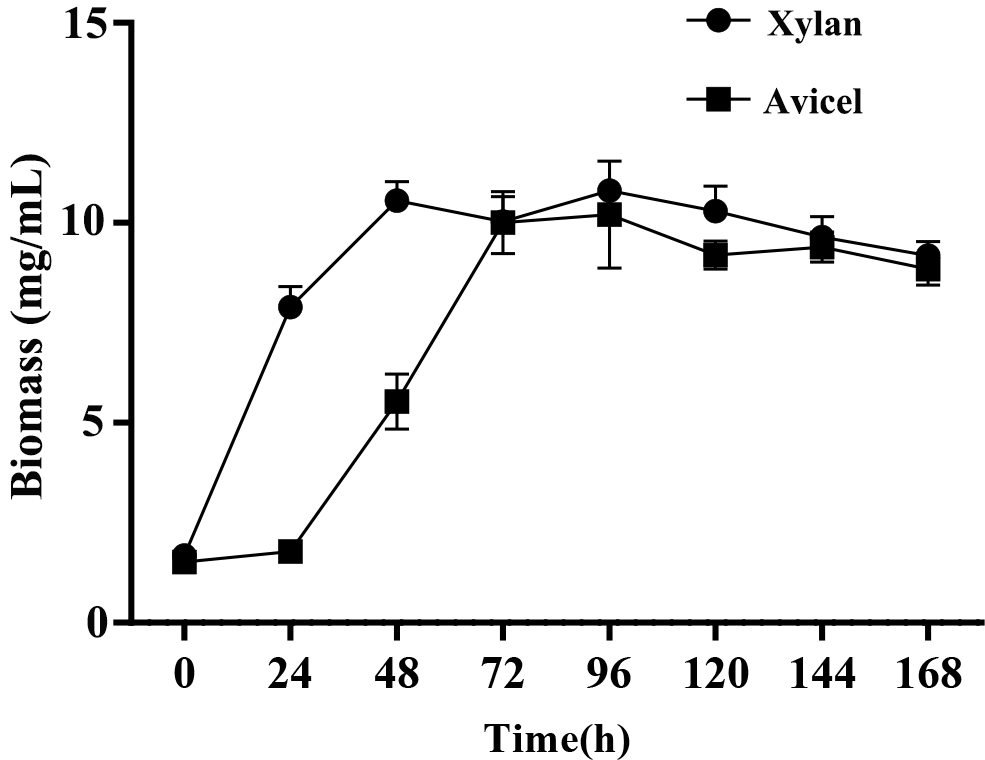
**
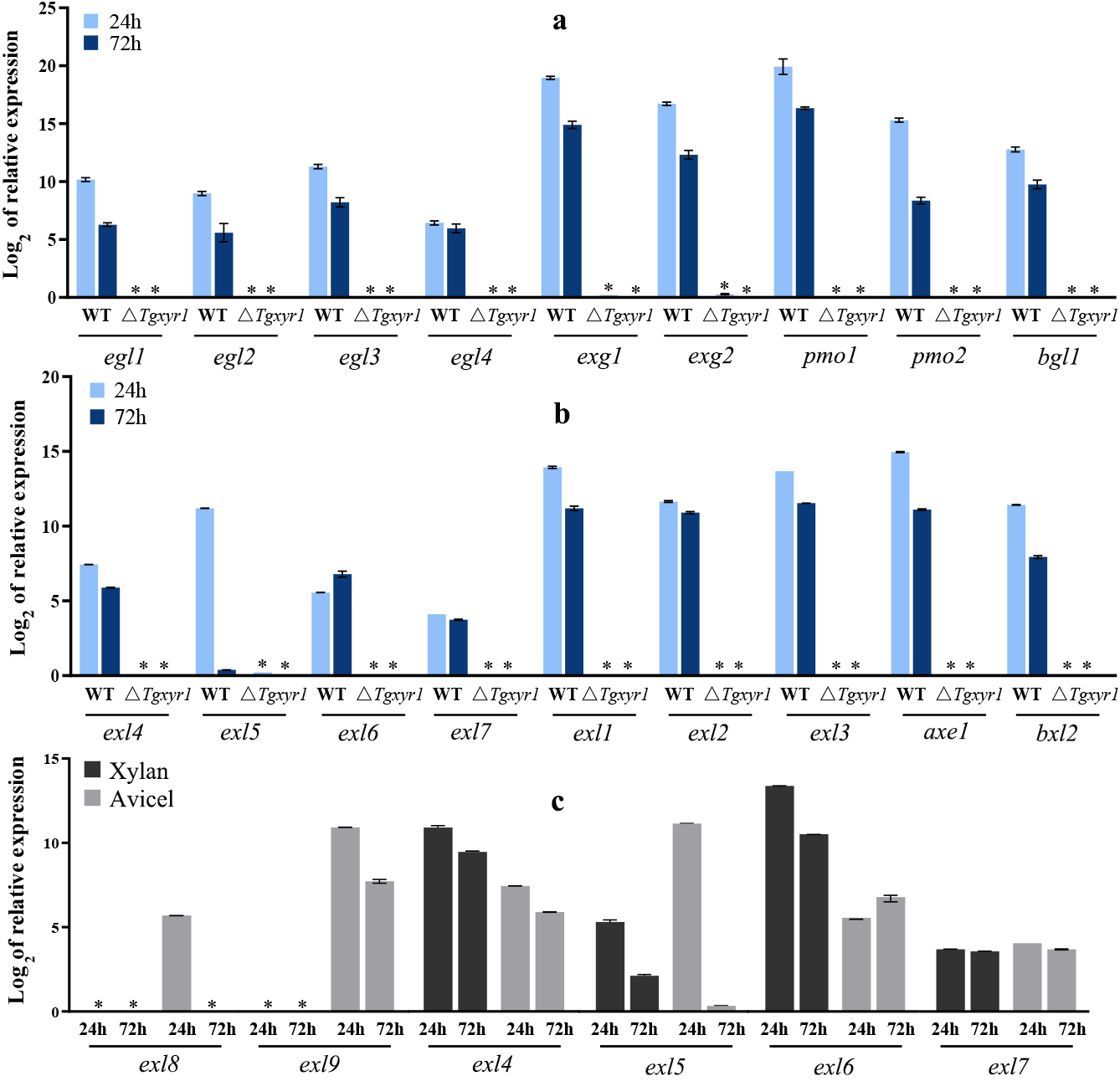
Fig S3. qPCR verification of cellulase and xylanase genes.** The expression levels of major cellulase(**a**) and xylanase(**b**) genes in NJAU4742 and *∆Tgxyr1* were detected by qPCR when induced by Avicel for 24 h and 72 h. Expression levels of GH10 (*exl8* and *exl9*) and GH11(*exl4*, *exl5*, *exl6* and *exl7*) xylanases were compared when strain NJAU4742 induced by Avicel and xylan for 24 h and 72 h (**c**). Error bars represent the standard deviation of three biological replicates.

**Fig S4. The growth curve of strain NJAU4742 when induced by Xylan or Avicel.** Strain NJAU4742 was incubated by glucose firstly, and then transformed into fresh MM medium containing 1% (w/v) xylan or 1% (w/v) Avicel for further incubation. Strain biomass was detected in the incubation time of 0 h, 24 h, 48 h, 72 h, 96 h, 120 h, 144 h and 168 h. Each sample had three replicates.

**Table S1 Primers used for gene knockout**

| **Primers** | | **5’ Sequence** | **3’ Sequence** |
| --- | --- | --- | --- |
| The donor DNA of UF+*hph*-EC+DF for *Tgxyr1* | UF | CTGTACTGTACGTGTTGGACGAC | GCCATATTGATGTAAGGTAGCTCTCAATAGGAGATGGCTGAACTGTGTG |
|  | DF | GGGTATTCCATCTAAGCCATAGTACCGATATCCCTGTGATGGTGTGTACG | GAAGCTCCAAGTCTTGTCTGAGC |
|  | Mutant detection | CCACGGCAATAAACTTGGTCG | CGTCTCAATCTTTTCTCGCGC |
| The donor DNA of UF+*hph*-EC+DF for *Tgclr2* | UF | AGCTTAGTGGGATGAACCTCATG | GCCATATTGATGTAAGGTAGCTCTCGTACCTCTGTTGGCCAGAGTG |
|  | DF | GGGTATTCCATCTAAGCCATAGTACCACAAAACCACACCGAAGACCC | AGTCAGCTCTTCCCTATGAGCC |
|  | Mutant detection | GGAGTATTACTGGTACGGGTGG | CTCTACCCATGACCTTCTCGC |
| The donor DNA of UF+*hph*-EC+DF for *Tgace1* | UF | GGGAGCAAAGATGGCGTCTTG | GCCATATTGATGTAAGGTAGCTCTCCCCTAGGTGGGGAAGATGTATGAG |
|  | DF | GGGTATTCCATCTAAGCCATAGTACCCCCGGTGATATCTTTGCCATTGTAC | GGAAGTAAGGCATGTCATCGATGC |
|  | Mutant detection | GTAACTAGCACGAGCTGCTAGC | AACTCTGTCCCAGAAGTGGAGAC |
| The donor DNA of UF+*hph*-EC+DF for *Tgace2* | UF | CATTATCGTCTACGGCAGGTGAG | GCCATATTGATGTAAGGTAGCTCTCAGTTTTCGATGAACCTCATTGGGAG |
|  | DF | GGGTATTCCATCTAAGCCATAGTACCCTGCAGCTCGAAATGGGAGATAG | GACGTGTGCATATAACCGGC |
|  | Mutant detection | AGCCGTCAATTTCATGCCG | CTCGTATGTACAGCTCGTACCG |
|  | *hph*-EC | GAGAGCTACCTTACATCAATATGGC | GGTACTATGGCTTAGATGGAATACCC |

**Table S2 Primers used for qPCR**

| **Gene** | **5’ Sequence** | **3’ Sequence** |
| --- | --- | --- |
| *exl8* | GACAAGCCTCCGTGAGCATTG | GCTATTCTCTGGCGTCACTTGG |
| *exl9* | ACATTCGTGCCAGGACAGTTTG | GCCATGTTGCTGTGCGTAATTG |
| *exl5* | TTGTCGGAGGCAAGGGATGG | CAGCCGTAGATGGAGAGGTAGC |
| *exl4* | CTACACTACTGGCGGCGATGT | CAGTGCTCCAACCAACTCCAAC |
| *exl6* | CTTCGGTGTCAACAGCGGAAC | ACGGTGCCTTGAGCAGGATAG |
| *exl7* | TACCGCACTACGACAGCAAGG | CAGCAGCGTTGGTCTGAAGTAG |
| *pmo2* | AGACATCACTCCGCTGGGTCA | ACGGTCCACTTGCTGCCTTG |
| *pmo1* | TCCTTGCCACCGCACTAACG | GCCGTCCAGCCTATCACAATG |
| *egl2* | GGCTGGCAAGATACGGAGACA | CCGTTGAAGCCATAGTAGAGCG |
| *egl1* | GCAATGCGGAGGTCAAGGATG | AGGTCGGCTTGGTTGTGGTAG |
| *exg2* | CAAGGTCGGTCTGGCAAGCA | TGTTGGCAGAAGGACGGATACC |
| *egl4* | TGACTGGAACTACCGCTGGATG | GGCACCACAAGTCGCTTCATC |
| *exg1* | CCACCAAGAAGTTGACCGTTGT | TTGATCGTGTTGCCAGTGTAGC |
| *bgl1* | GCTGGCAACGATGTCGAGATG | GAGCGAAGCACACGGGAAAC |
| *exl2* | CAGATTAGTCCCAACGCCCAAG | CATCACAGCAGGTAAGCCTCAG |
| *exl1* | CGGAACCGTATCGGATCTGGAG | GACTGCCTGTCTCGTGAACCA |
| *exl3* | AGAACTCCAATGGCGACAATGC | TGCGTACAGAACCTGGTCTAGC |
| *bxl2* | CAAGAAGGCTGGCTACAAGGTC | GCGAAGATGATGGCGTCAGAC |
| *axe1* | GCTCCTCCGCTACTGTTGTGA | TGGCTACGGCGTTGATACCTG |
